# Progress in methods for rare variant association

**DOI:** 10.1186/s12863-015-0316-7

**Published:** 2016-02-03

**Authors:** Stephanie A. Santorico, Audrey E. Hendricks

**Affiliations:** Department of Mathematical and Statistical Sciences, University of Colorado Denver, Denver, CO 80217-3364 USA

## Abstract

Empirical studies and evolutionary theory support a role for rare variants in the etiology of complex traits. Given this motivation and increasing affordability of whole-exome and whole-genome sequencing, methods for rare variant association have been an active area of research for the past decade. Here, we provide a survey of the current literature and developments from the Genetics Analysis Workshop 19 (GAW19) Collapsing Rare Variants working group. In particular, we present the generalized linear regression framework and associated score statistic for the 2 major types of methods: burden and variance components methods. We further show that by simply modifying weights within these frameworks we arrive at many of the popular existing methods, for example, the cohort allelic sums test and sequence kernel association test. Meta-analysis techniques are also described. Next, we describe the 6 contributions from the GAW19 Collapsing Rare Variants working group. These included development of new methods, such as a retrospective likelihood for family data, a method using genomic structure to compare cases and controls, a haplotype-based meta-analysis, and a permutation-based method for combining different statistical tests. In addition, one contribution compared a mega-analysis of family-based and population-based data to meta-analysis. Finally, the power of existing family-based methods for binary traits was compared. We conclude with suggestions for open research questions.

## Background

Rare variants have increasingly become a focus in studies of complex traits. There are many reasons for this increasing interest. Accessibility, both in cost and technology, of next-generation sequencing has led to the discovery of a plethora of rare variants. Nelson et al. [1] estimated that 95 % of variants were rare with a minor allele frequency (MAF) of less than 0.5 %. This is in stark contrast to previous research suggesting that nearly one-third of variants have a frequency below 5 % [2]. Furthermore, evolutionary theory suggests that deleterious variants are selected against and thus should be rare [3]. Recent research has supported this theory, observing that a large proportion of deleterious variants are indeed rare [4, 5]. Despite the effects of this purifying selection, the 1000 Genomes project estimates that individuals carry 76 to 190 rare nonsynonymous variants predicted to be deleterious [6].

A more contentious argument for focusing genetic research on rare variants pertains to the so-called phenomenon of “missing heritability”. Genome-wide association studies (GWAS) have successfully identified numerous common variants associated with complex traits; however, the common variants tend to have relatively small effects and explain only a fraction of the overall heritability [7]. Human height serves as an excellent example with estimates of heritability near 80 %. GWAS variants with genome-wide significant associations explain only approximately 5 % of overall variation in height whereas models that use all high-quality GWAS variants with a MAF of more than 1 % explain approximately 45 % of the variation [8, 9]. Even though the latter is a substantial improvement, it is still well shy of 80 %.

There is emerging evidence that rare variants are involved in complex disease, including Alzheimer disease [10], lipids and coronary artery disease [11], irritable bowel disease [12], prostate cancer [13], and many others [11, 14–16]. Despite these encouraging results, many studies continue to be underpowered to detect association to disease-associated rare variants. Continued development of methods is needed to help increase the power to detect these associations. This is particularly true given that tests of individual rare variants are underpowered without exceptionally large sample sizes [14]. Combining variants based on a gene or region is a popular strategy. Other strategies for improving power for detecting rare variants include using family samples or isolated populations to increase the frequency of a variant that is rare in the general or nonisolate population [17, 18]. Ascertaining phenotypic extremes can also increase the likelihood of sampling individuals with disease-associated rare variants, thus increasing the power of rare variants tests [19, 20]. Finally, incorporating biological knowledge and genomic annotation to exclude, or downweight, variants in analyses is also an effective strategy, focusing tests on variants more likely to be deleterious [21].

Here, we provide a summary of the current literature with respect to the association of rare variants and ways to increase the power of these tests. We then provide results from the Collapsing Rare Variants Working Group of Genetic Analysis Workshop 19 (GAW19) and conclude with recommendations and open problems.

### Current literature

Although there is no formal definition for a rare variant, variants with a MAF between 5 % and 50 % are generally considered common. Variants with a MAF in the range of 1 % to 5 % [15] or 0.5 % to 5 % [22] are considered low frequency or less common. Rare variants have a MAF falling below these ranges, whereas a private variant is specific to probands and their relatives.

### Basic association models for collapsing rare variants

The 2 major types of methods for collapsing rare variants within a meaningful genetic region, such as a gene, consist of burden tests and variance component tests. Burden tests measure the burden of variants within a genetic region and range from a simple indicator of whether a genetic region contains at least 1 rare variant (eg, CAST [cohort allelic sums test] [23]), to a sum of the rare variants within a region (eg, ARIEL [accumulation of rare variants integrated and extended locus-specific test] [24], CMC [combined multivariate and collapsing] [25], and MZ [Morris and Zeggini] [26]), to a weighted sum of the rare variants in a region (eg, WSS [weighted sum statistic] [27] and aSum [data-adaptive sum] [28]).

A general formula for the burden of rare variants within a region is shown in Eq. (1).

1 $$ {B}_i={\sum}_{m=1}^M{G}_{i,\kern0.15em m}{w}_m $$

where *G*_*i*,*m*_ is the genotype coding based on a genetic model (eg, recessive, dominant, or additive) for individual *i* and variant *m*, *w*_*m*_ is the weight for variant *m*, and *M* is the total number of variants in the region. This model is applicable to nearly all burden tests mentioned above. For example, the CAST is often written in terms of an indicator function such as ${B}_i=I\left({\sum}_{m=1}^M{G}_{i,m}{w}_m\right)$ where *G*_*i*,*m*_ is either 1 or 0, depending on whether the subject has or does not have variant *m*, respectively, and *w*_*m*_ = 1. However, we can use Equation (1) directly by making ${w}_m=\frac{1}{{\displaystyle {\sum}_{m=1}^M}{G}_{i,m}}$ to ensure that *B*_*i*_ is 1 for subjects who have at least 1 variant in the gene and 0 otherwise. For a simple count of the number of variants within a region, a weight of 1 for each variant is used. Others have proposed more informative weights. Madsen and Browning [27] proposed a weight that increases as MAF decreases while Asimit et al. [24] weighted genotypes by their quality.

Even though several tests were first developed outside of the regression framework [23, 25, 27], nearly all can be easily implemented in a generalized regression framework (Eq. 2) by incorporating ${B}_i$ as a covariate in the regression model. This greatly generalizes the statistical framework allowing for many types of outcome variables (eg, continuous, binary, survival, etc.), and the incorporation of additional possible confounders and covariates:

2 $$ f\left(\mu \right)={\gamma}_0+\boldsymbol{\gamma} \boldsymbol{\hbox{'}}\boldsymbol{X}+\beta B $$

where *f*(*μ*) is a function that links a linear combination of the predictors and the mean, *μ*, of the outcome (eg, disease or trait); *γ*_0_ is the intercept; ***γ*** ' is a vector of parameters for the covariates, ***X***; *β* is the regression parameter for the burden of rare variants within a region, *B*; and bolded symbols denote a vector. For a quantitative trait *f*(*μ*) = *μ* is used within a linear regression framework, and for a qualitative trait *f*(*μ*) = logit(*μ*) is typically used within a logistic regression framework. Although several test statistics can be implemented within the generalized regression format, we focus on the score statistic, *U*, testing whether *β* = 0. The burden score statistic is shown in Equation (3) and under the null hypothesis of no association has a chi-square distribution with 1 degree of freedom (df). Note that the burden score statistic can be written as a weighted sum of the marginal score statistics, *S*_*m*_, for each genetic variant where, ${S}_m={\sum}_{i=1}^n{G}_{i,m}\left({y}_i-{\widehat{\mu}}_i\right)$ for *n* individuals, with ${\widehat{\mu}}_i$ being the estimated mean for individual *i*, which includes the effects of covariates as estimated through generalized regression.

3 $$ {U}_{burden}={\left[{\displaystyle \sum_{i=1}^n}{B}_i\left({y}_i-{\widehat{\mu}}_i\right)\right]}^2={\left[{\displaystyle \sum_{m=1}^M}{w}_m{\displaystyle \sum_{i=1}^n}{G}_{i,m}\left({y}_i-{\widehat{\mu}}_i\right)\right]}^2={\left[{\sum}_{m=1}^M{w}_m{S}_m\right]}^2\sim {\chi}_1^2 $$

As marginal score statistics can first be calculated on each variant, this alternative form lends itself nicely to extensions such as meta-analysis as described later.

Instead of calculating the burden of variants within a genetic region, variance component tests (eg, sequence kernel association test [SKAT] [29], C-alpha [30] and SumSqU [31]) evaluate the similarity of the variants within the region. Simply, we expect the distribution of variants to be more similar for subjects with similar trait values than for subjects with different trait values. Like with the burden test, a general equation for the score statistic of the variance component test can be written and is shown in Eq. (4).

4 $$ {U}_{VC}={\sum}_{m=1}^M{\left({w}_m{S}_m\right)}^2 $$

where *S*_*m*_ is the previously defined marginal score statistic. *U*_*VC*_ follows a mixture chi-square distribution. Because the marginal score statistic is squared, both negative and positive effects can be included in the statistic. This is a notable advantage of variance component tests over burden tests, for which effects of different directions can cancel each other out. For both C-alpha [30] and SumSqU [31], the weights equal 1. C-alpha is further restricted to scenarios where the phenotype is dichotomous, and there are no covariates. The SKAT statistic [29] is identical to *U*_*VC*_, accommodating a variety of weights; as such, C-alpha and SumSqU are special cases of SKAT.

Burden tests tend to be most powerful when the majority of variants have an effect in the same direction [25, 29, 32]. Variance component tests are more powerful when the variants have different effects (ie, many variants with no effect or effects in opposite directions) [29, 32]. To combine the different strengths of the burden and variance component tests, Lee et al. [32] developed an optimal unified approach called the SKAT-O, where the burden and SKAT tests are combined with a weighting parameter, *ρ* (Eq. 5). Note that the optimal test is equivalent to the burden test and SKAT (ie, variance component test) when *ρ* is 1 or 0, respectively.

5 $$ {U}_{optimal}=\left(1-\rho \right){U}_{SKAT}+\rho {U}_{burden},\ 0\ \le\ \rho\ \le\ 1 $$

Others have explored combining the burden and variance components tests as well [33, 34]. Finally, more recently the EC test [35] was developed under a Bayesian framework with an alternative hypothesis prior that gives a higher probability to only 1 causal variant per genetic region.

Here, we provide a basic overview of general methods; others have done this as well in more detail [22]. In addition, Derkach et al. [36] have provided an excellent review and comparison (both empirical and theoretical) of existing methods. Important conclusions and results include: weighting variants inversely to the MAF does not always increase power even under scenarios where rare variants where simulated to have a larger effect; as the sample size increases the variance component statistic tends to have a higher power than the burden statistic; uniformly optimal tests are difficult to achieve in practice.

### Incorporating additional information

There have been many extensions to the basic frameworks and models to include and account for additional information. Various weights can be defined based on the MAF [27], quality of genotype calls [24], previous evidence for association, direction of effect (eg, aSum [28]), evolutionary conservation (eg, phastCons [phylogenetic analysis with space/time models conservation] [37], and GERP [genomic evolutionary rate profiling] [38]), probability of being functional, and likelihood of being deleterious. There exists several algorithms/software that predict whether a variant is likely to be deleterious, including CONDEL (consensus deleteriousness) [39], SIFT (sorting intolerant from tolerant) [40], PolyPhen (polymorphism phenotyping) [41], CADD (combined annotation-dependent depletion) [42], and several others (see Castellana and Mazza [43]). Although the predictions of these programs can differ greatly [39, 43, 44], variants that have consistent predictions of either being benign or deleterious across all programs may be more likely to be truly benign or deleterious. Variants can be removed entirely from the model by using a weight of 1 and a weight of 0 for variants fulfilling or not fulfilling a requirement or threshold, respectively. It is often difficult to know the true or best threshold to use when determining which variants to include in the model. Adaptive methods implement the region-based methods over a variety of thresholds (such as various MAF thresholds) and then adjust for multiple comparisons using permutation [28, 45].

It is worth emphasizing that the proportion of variants in the collapsing test with association to the outcome is directly related to the power [46, 47]. As such, choosing which variants to include is extremely important. When choosing variants, various factors should be considered such as the likely penetrance of the variants, the prevalence of the disease or trait, and the predicted deleteriousness of the variants. As discussed in the previous paragraph, instead of weighting variants, only a subset of variants can be kept, such as those predicted to be deleterious or to result in loss of function.

Once a gene or region has been identified as being associated to a disease or trait, an important next step is to identify the causal variants within the region. Experimental studies to determine the functional effects are often costly both in effort and money. In a recent paper, Ionita-Laza et al. [48] proposed and compared 2 methods to identify likely causal variants within gene regions.

Unlike rare variants, the parameter estimation for common variants is generally stable. Including disease-associated common variants within a gene region could help to identify genetic regions associated with a trait as well as to help determine if a collapsed set of rare variants produces an independent signal above that from the common variants. Determining which common variants to include in the model is not always straightforward as too many variants will dilute the signal and decrease the power by using up valuable degrees of freedom, while including too few variants may miss a signal all together. Penalized regression methods, such as LASSO (least absolute shrinkage and selection operator), have been proposed [49] as well as an extension to the SKAT framework that incorporates common variants [50].

More recently, methods have been developed to compare the observed number of filtered variants within a genetic region to that expected genome- or exome-wide [51] or expected by an estimated mutation rate [52]. These methods are most often implemented in a case-only framework, and are thus sensitive to the estimates of comparison (eg, genome-/exome-wide averages, mutation rates, etc.). These methods are discussed further below.

### Study design considerations

Although sequencing costs continue to decline, the cost of sequencing continues to impose a limit on the number of samples that can be sequenced. There is increasing evidence that the power to detect an association to rare variants is low regardless of the type of test or statistic used [46, 47]. As such, study design is of utmost importance and includes, among others, family-based, trio, case-only, case–control, and population cohort designs.

Study design affects the power and generalizability of the study. Certain study designs may increase the power to detect an association in certain situations while decreasing the ability to detect other genetic associations. For instance, sampling families with a particular rare disease increases the likelihood of observing multiple copies of the causal rare variant, thus increasing power to detect an association to that particular variant [53]. However, this sampling framework may reduce the number of detected genetic variants, making it more difficult to discover the variety of genetic variants that would be seen when sampling the general population. Recently, several methods have been developed or extended to accommodate related samples [54, 55]. Probably the most widely used of these is famSKAT (family-based sequence kernel association test) developed by Chen et al. [56], which extends SKAT by using a linear mixed effects model to account for the family structure in tests of quantitative traits. For GAW19, Wang et al. [57] studied the type 1 error and power of current family-based methods for rare variant association tests for dichotomous phenotypes. It is also important to note that valid permutation to assess significance in the context of dependent samples (such as with related samples or population stratification) is not straightforward. Others have explored permutation in this setting and have proposed modified permutation procedures [58].

For extremely rare, highly penetrant disorders, researchers have had success sequencing a set of cases [59, 60] or trios where the offspring has an extremely rare disorder and the parents are unaffected [61–63]. Specific software exists for detecting de novo mutations within trio designs [64]. For more complex and common diseases or traits, study designs such as a case–control or population-cohort are often used [21, 65, 66]. Although many case–control studies are retrospective, few incorporate the retrospective ascertainment of the sampling design into the statistical framework. Such methods were included in GAW19 contributions [57, 67]. Unfortunately, detecting rare genetic associations in complex diseases has continued to prove difficult and much larger sample sizes are needed to achieve adequate power. Some study designs use extreme sampling either of cases [68] or of quantitative phenotypes [21] to increase power. For complex traits, extreme sampling can lead to an increase in the number of rare variants detected and subsequently an increase in power [69]. However, not accounting for the trait-dependent sampling when analyzing quantitative traits can lead to biased estimates, inflated type 1 error, and even a decrease in power [70]. In 2013, Barnett et al. [69] and Lin et al. [70] each developed novel statistical methods to appropriately analyze quantitative traits with extreme sampling study designs.

Within the study design of sequencing a unique and homogeneous set of cases, case-only statistical frameworks exist for detecting exceedingly rare or de novo and highly penetrant variants [51, 52]. Statistical frameworks also exist to incorporate external population controls with the unique set of cases in a case–control analysis [71], although more research in this area is needed.

As previously discussed, most methods can be expressed within a regression framework. Many of the burden methods are within a generalized linear regression framework while the variance component methods, such as SKAT, are implemented within a mixed effects regression model. The original regression frameworks of these methods required large enough sample sizes to reach an asymptotic distribution of the test statistics and independent observations. Few methods have been developed specifically for small samples, although Lee et al. [72] extended SKAT for use with small sample sizes.

### Meta-analysis

Meta-analysis of test statistics across multiple studies is widely used in GWAS and other genetic studies of common variants to replicate, confirm, and find new associations. Meta-analysis is arguably even more important for studies of rare variants where extremely large sample sizes are important for achieving adequate power. Many simple meta-analysis frameworks that combine information about the test statistic or *p* value (such as Fisher’s and Z-score methods [73]) can be applied to test statistics from current region-based methods. (Although it should be noted that, as there is no direction of effect for variance component tests, only weights based on sample size and not direction of effect can be incorporated into Z-score meta-analysis for variance component tests.) Although simple and easy to implement, these meta-analysis methods do not account for different variants that may be included in the region-based statistics for each study.

Lee et al. [74] developed a meta-analysis framework for rare variants that achieves nearly identical empirical power as analyses based on combined individual level data (sometimes called mega-analysis). This framework uses single-variant score statistics and the corresponding between-variant covariance matrix. Importantly, the framework allows for variants to be monomorphic (ie, the alternate allele is not seen) in some of the individual studies. To be included in the meta-analysis statistic, a variant only has to be polymorphic in at least 1 study. Further, meta-analysis has other advantages such as easier sharing of data (given consent or computational barriers to sharing raw data) and controlling for potential confounders or population stratification specific to each study. For instance, one study may adjust for 5 principal components whereas another study may adjust for 3 principal components and recruitment center. In addition to being able to include different study-specific covariates, one can also further account for possible heterogeneity in study statistics in the meta-analysis statistic itself as described below.

Here, we briefly outline Lee et al’s [74] meta-analysis framework. If we define the single-variant (ie, marginal) score statistics as *S*_*k*, *m*_ for study *k* and variant *m*, we can then rewrite the burden score statistic as a combination of the single-variant statistics over all studies:

$$ {U}_{burden\_ meta}={\left[{\varSigma}_{m=1}^M{\varSigma}_{k=1}^K{w}_{k,m}{S}_{k,m}\right]}^2. $$

We can also square the single-variant score statistics summed over the studies and then summed over variants to produce a meta-analysis score statistic for the variance component region test:

$$ {U}_{VC\_ meta\_hom}={\varSigma}_{m=1}^M{\left({\varSigma}_{k=1}^K,{w}_{k,m}{S}_{k,m}\right)}^2. $$

The above variance component statistic requires the additional assumption of homogeneous genetic effects across all studies. If we believe that the genetic effects are instead heterogeneous, the meta-analysis score statistic for the variance component region test can be written as follows:

$$ {U}_{VC\_ meta\_het}={\varSigma}_{m=1}^M{\varSigma}_{k=1}^K{\left({w}_{k,m}{S}_{k,m}\right)}^2. $$

If we believe the heterogeneity can be isolated to clusters of studies, such as ethnicity, the statistics can be combined, first over the studies in each cluster and then over each cluster and marker. Note that the burden and variance component meta-analysis test statistics can be combined in an optimal way similar to that shown for single studies in Equation (5). More details are in Lee et al. [74]. Others have explored meta-analysis for rare variants as well [75].

## Contributions from the collapsing rare variants working group

GAW19 provided real human sequence and phenotype data for data sets of Mexican American families and unrelated individuals. In addition, 200 simulated data sets were provided based off the real sequence data for phenotypes with true underlying genetic associations as well as a null polygenic trait. Family data (for 959 individuals in 20 pedigrees) consisted of whole genome sequencing calls and GWAS single-nucleotide polymorphisms (SNPs) for odd-numbered chromosomes, as well as longitudinal real phenotype data for systolic and diastolic blood pressure, age, sex and indicators of hypertension, antihypertensive medication use, and cigarette smoking, collected at up to 4 time points. Family data also included genome-wide measures of gene expression for a smaller set of individuals; however, no contribution in our group utilized this data nor did any contribution utilize the longitudinal nature of the data. The data set of 1943 unrelated individuals contained exome sequence calls and the same phenotypes as the family data, at a single time point. More detailed information on the GAW19 data sets is available in Blangero et al. [76].

The 6 contributions from the Collapsing Rare Variants Working Group of GAW19 extend upon the current literature and reflect varied goals, including the creation of new statistical tests, developments of meta-analytic techniques and a comparison of existing statistical tests. Table 1 provides overall characteristics of each contribution.

**Table 1:** Contributions from the GAW19 Collapsing Rare Variants working group

Goal	Reference	Trait	Data type	Statistic type
New statistic	Green et al. [77]	Quantitative	Family simulated	Combined burden and variance component
	Jadhav et al. [78]	Dichotomous	Unrelated, simulated	Burden and Variance component
	Zhu et al. [67]	Quantitative	Family simulated	Variance component
Meta-analysis	Katsumata and Fardo [81]	Quantitative	Family and unrelated simulated	Variance component
	Wang et al. [82]	Quantitative	Family and unrelated real data	Variance component, haplotype model
Method comparison	Wang et al. [57]	Dichotomous	Family simulated	Burden, variance component and an optimal combination

### New statistics

Green et al. [77] developed a general framework for combining different statistical tests of association of rare variants with a continuous trait in family-based studies. A linear mixed model was used to derive residuals by adjusting for covariates as well as a random effect for familial correlation. These residuals were then permuted to create data sets reflective of the null hypothesis of no association, allowing for the derivation of empirical *p* values that combine information over a set of rare variant tests yielding a single overall test of association. In the Green et al. formulation [77], evidence was combined over 4 burden tests and 4 variance–component tests representing different powers of the marginal score statistics (*U, U*^*2*^*, U*^*3*^ and *U*^*4*^) as well as over 2 weight functions, one based on the Beta distribution [29], and the other based on the inverse standard deviation of the allele count [45]. With increasing powers of the marginal score statistics, the contribution of noncausal variants to the overall statistic is lessened, and the use of the Beta distribution more severely down weights common variants compared to those based on the inverse standard deviation of the allele count. By utilizing all combinations of weight function with powers of the score statistic, a variety of models are included within the test. However, given the permutation framework, their method can be generalized to any set of statistical tests. In evaluating their method, Green et al. [77] focused on the GAW19 simulated data set of 30 genes on chromosome 3 that have at least 1 casual variant; type 1 error and power were estimated for the combined approach, as well as for each of the 4 burden tests and 4 variance-component tests. Type 1 error was controlled at the 0.05 level based on the null trait, Q1, provided with the simulated data. The combined approach consistently yielded intermediate power relative to the power of the four burden and four variance-component tests. Given there is no single best test and that the optimal statistic is unknown a priori, the combined approach allows for proper control of type 1 error and is an approach that is robust to differing genetic architectures. Further research is needed to determine an optimal combination of tests, ones that are uncorrelated, reflecting different patterns of association, and that maximize power.

Zhu et al. [67] derived a score test, OW-score, based on the retrospective likelihood for a continuous trait formed by conditioning on observed phenotypes. The resulting test is a function of a weighted combination of genotypes over the variants included in the test, where the weighting is derived to maximize the score statistic. Power for the OW-score method was compared to that of famSKAT [56] for the 14 genes with the largest simulated signal for diastolic blood pressure in the GAW19 data at a significance level of 0.05. Only 4 genes, yielded power that was greater than 40 % for either method; of these, the OW-score test was more powerful for 3 genes and the famSKAT was more powerful for 1 gene. It is important to note that the distribution of the weights by MAF differs between the OW-score test and famSKAT, with famSKAT more highly weighting variants with a MAF within (0.01, 0.05). This aligned with the simulation results and performance of the OW-score test compared to famSKAT: when causal variants fell within this MAF range, the famSKAT was more powerful than the OW-score method. Thus, more research is needed to determine if the retrospective nature of the OW-score test or the varied weighting structure is leading to increased power in certain scenarios.

Jadhav et al. [78] used a method, from the branch of statistics called *functional data analysis*, which is based on analysis of curves, surfaces, or functions [79]. Specifically, a functional analysis of variance (ANOVA) model compared the difference in the genetic structure of a genomic region between cases and controls. To do so, a continuous function was fit to each individual’s genotype using cubic B-splines over a 30-kb region, and the resulting mean function was compared between cases and controls using an ANOVA test. Results were compared with a burden test that weighted minor allele counts by the inverse standard deviation for the minor allele count in controls [27], as well as to a burden test that incorporates linkage disequilibrium through a genetic covariance matrix [80]. Simulations were conducted for a 1.4-Mb region of chromosome 3 where causal variants were randomly selected to be 1 % to 50 % of the region, and phenotypes were simulated using both unidirectional and bidirectional effects. The functional ANOVA test had greater power, up to 0.135 higher, compared to the burden test (with or without incorporation of linkage disequilibrium) over all but 1 scenario, in which 50 % of the variants in the region were causal and with unidirectional effect. In this scenario, power was comparable.

### Developments in meta-analysis

Katsumata and Fardo [81] applied the famSKAT statistic to each of the GAW19 family- and population-based data sets, as well as to the combined set of data, resulting in a mega-analysis. These 3 analyses were compared against a meta-analysis of the family- and population-based data sets for the 15 most causal genes influencing each of diastolic and systolic blood pressure in the GAW19 simulation model (23 genes in total, given overlapping causal genes). They found that mega-analysis could be substantially more powerful than meta-analysis (*NRF1*, *LEPR*, *LRP8*, *GAB2* with systolic blood pressure [SBP]) with meta-analysis resulting in discernibly higher power compared to mega-analysis for only one of the top genes (*TNN* with both SBP and diastolic blood pressure [DBP]). However, when the power to detect association to a gene-region was considerably lower within the family-based sample versus the population-based sample, the power of the mega-analysis was much lower than the analysis based on the population-based sample alone, while the meta-analysis had a less-severe power loss. This suggests that the mega-analysis may be better when there is sufficient power to detect an association in both samples, but a meta-analysis might be more suited to situations where one study is underpowered and/or there is heterogeneity in the genetic associations between study samples. Both meta-analysis and mega-analysis indicated elevated type 1 error with estimates based on the 200 simulated data sets of the null trait Q1 ranging from 0.055 to 0.130 and 0.050 to 0.135, respectively, for the 23 genes.

Wang et al. [82] also considered a meta-analysis of the famSKAT statistic applied to the family- and population-based data sets for DBP. These results were compared to a meta-analysis of results from a haplotype-based association model. For haplotype analysis, a mixed linear model was fit, allowing for covariates, fixed effects of haplotypes (with haplotypes with frequency of less than 0.5 % collapsed into 1 group) and random components for family structure and error. Haplotypes were coded using dosages estimated from genotypes using the expectation–maximization algorithm. Models were fit separately for the family-based and population-based samples, and the weighted-least squares method of meta-analysis was followed by a Wald test of equal haplotype effects. Type 1 error for the haplotype model was found to be elevated for genes with more than 14 haplotypes; hence, results on the real data set were given for only genes with fewer than 14 haplotypes. None of the genes were significant for famSKAT after correcting for multiple testing; however, multiple genes did achieve statistical significance using the haplotype model indicating a potentially more powerful method for association testing. As these results are from real data, further study is needed to understand relative performance of the 2 methods over a range of models.

### Method comparison

Finally, Wang et al. [57] compared existing family-based methods for binary traits including the rare variant transmission disequilibrium test (RV-TDT) [55], the generalized estimating equations–based–kernel association (GEE-KM) test [83], an extended CMC test for pedigree data known as PedCMC [84], a gene-level kernel and burden association tests for pedigree data (PedGene) [80], and the family-based rare variant association test (FARVAT) [85]. Through simulation based on the 6 genes with the largest effects on both simulated SBP and DBP, they found that the FARVAT method based on optimal weights (that adaptively use the data to combine burden and variance component tests) was more powerful than the PedCMC, GEE-KM, or any of the RV-TDT tests. The power for the PedGene method was comparable with that of FARVAT; however, FARVAT required substantially less computing time. Based on dichotomization of the simulated null trait Q1 to correspond to a prevalence of 22.6 %, type 1 error was demonstrated to be deflated for the RV-TDT and inflated for the GEE-KM test, while the PedCMC, PedGene, and FARVAT had reasonable control of type 1 error across a range of significance levels.

## Discussion and conclusions

Over the last 10 years, there has been considerable methods development for association tests of rare variants. Tests have been proposed that are ideal for unidirectional and bidirectional effects, as well as an optimized combination of the 2 types of effects. Methods have been proposed for binary as well as normally distributed traits, for population-based and family studies. Most tests allow for the use of different weighting schemes (eg, based on MAF or genomic annotation), and meta-analysis procedures have also been developed.

Contributions to the GAW19 Collapsing Rare Variants group expanded upon the literature in several ways. Green et al. [77] provided a method that could be used to combine any collection of statistics for rare variant association. This is particularly important given there are numerous types of annotation that could be used as weights and these weights could be implemented in a burden model, variance component model, or combination of the 2 models. While an oft-used strategy is to conduct all tests separately, the method proposed by Green et al. would allow for an empirical combination in a statistically rigorous framework while controlling for total type 1 error.

New statistical tests were developed to allow for a retrospective likelihood based on optimized variant weights [67] and to incorporate genomic structure into the test of rare variants [78].

Katsumata and Fardo [81] provided guidance regarding design and meta-analysis. Based on GAW19 simulated data, they found that mega-analysis generally led to higher power than a meta-analysis; however, if there were large differences in power between the family-based and population-based studies, a mega-analysis could have power less than that of the studies being combined, meta-analysis was less affected by this scenario. Wang et al. [82] compared meta-analysis based on haplotypes to meta-analysis based on famSKAT statistics, demonstrating the 2 approaches to be complementary by detecting associations to different genes.

Finally, Wang et al. [57] compared existing family-based methods for rare variant association to binary traits and demonstrated PedGene and FARVAT to be powerful methods for rare variant association, with FARVAT being more computationally efficient.

Although much work has been done, there are still many open research areas pertaining to the analysis of rare variants. We mention a number of these areas; however, this list is by no means exhaustive. For example, great care has been taken in studies of common variants to control for population substructure. These have included the use of genetic principal components, genomic matching and linear mixed models; see, eg, the review by Price et al. [86]. Given rare variants are confounded with population ancestry, it is not clear how to best control for this substructure. Although there has been some work in this area in showing that population substructure is indeed different for common and rare variants [87, 88], more work, especially in method development, is needed.

It often makes sense to focus on the gene region as the unit for collapsing methods, especially given analyses within the coding regions of the genome. However, GWAS associations are often in intergenic regions, and there is building evidence that much of the noncoding region of the genome is indeed functional [89, 90]. Thus, there is an interest in testing noncoding regions of the genome for association with rare variants and how best to define the regions is an open question. A sliding-window based approach is often used to group regions of the genome for testing. There are many additional questions when using sliding windows such as number, size of window, and size of overlap between windows. Genomic windows will need to be large enough to capture the causal region without being too large so as to include too much noise. There is likely to be a tradeoff between multiple testing adjustments necessary to account for many small windows versus the potential power loss from using fewer windows that are too large. In addition, as the functionality of the noncoding regions continues to be discovered and defined, it is likely that there will be useful information to use when building or defining the windows or meaningful genetic units within the non-coding regions.

As we have detailed, methods exist for incorporating genomic annotation as weights in region based methods. The choice of the best weight and, in fact, which information to consider at all, remain somewhat open questions. Currently, MAF, functionality, consequence, evolutionary conservation and many other metrics can be used as weights and the list continues to grow, especially in non-coding regions as functional research continues at a rapid pace. Thus, there is a need to further develop efficient methods of deriving the most appropriate weight. This can be done to some extent through the adaptive methods discussed previously. However, the adaptive methods, which often rely on permutation, may become computationally infeasible given the increasing amount of information on which to weight, increasing sample size, and analysis on the entire genome. Thus, there will continue to be a need for computationally efficient methods of determining the weights while retaining the appropriate type 1 error.

To date, most collapsing of rare variants is done on a contiguous region of the genome, whether it is a gene or a genomic window. Alternative approaches include the use of pathways or gene sets developed, for example, from expression studies or protein-protein interaction studies. Recent studies have found some success with this approach [91], but more research is needed.

Finally, given the continued struggle to adequately power studies of rare variants, more work is needed on ways to improve power. One approach is to continually increase the sample size of the studies, perhaps through including publically available population controls. Another, perhaps more feasible approach may require refocusing the phenotype through use of multidimensional phenotypes or homogeneous subphenotypes.

Given this relatively brief discussion of remaining areas of research for the association of rare variants, there is little doubt that this will continue to be an active area of research for several more years.
